# Comparison of Four-Dimensional Flow MRI, Two-Dimensional Phase-Contrast MRI and Echocardiography in Transposition of the Great Arteries

**DOI:** 10.1007/s00246-023-03238-2

**Published:** 2023-07-24

**Authors:** Evangeline G. Warmerdam, Jos J. M. Westenberg, Michiel Voskuil, Friso M. Rijnberg, Arno A. W. Roest, Hildo J. Lamb, Bram van Wijk, Gertjan T. Sieswerda, Pieter A. Doevendans, Henriette ter Heide, Gregor J. Krings, Tim Leiner, Heynric B. Grotenhuis

**Affiliations:** 1 Department of Cardiology, University Medical Center Utrecht, Heidelberglaan 100, 3584 CX Utrecht, The Netherlands; 2 Department of Paediatric Cardiology, Wilhelmina Children’s Hospital, Utrecht, The Netherlands; 3 Department of Radiology, Leiden University Medical Center, Leiden, The Netherlands; 4 Department of Cardiothoracic Surgery, Leiden University Medical Center, Leiden, The Netherlands; 5 Department of Paedidatric Cardiology, Leiden University Medical Center, Leiden, The Netherlands; 6 Department of Congenital Cardiothoracic Surgery, Wilhelmina Children’s Hospital, Utrecht, The Netherlands; 7 Netherlands Heart Institute, Utrecht, The Netherlands; 8 Department of Radiology, University Medical Center Utrecht, Utrecht, The Netherlands

**Keywords:** Congenital heart disease, Advanced cardiac imaging, Magnetic resonance imaging, Transposition of the great arteries

## Abstract

Pulmonary artery (PA) stenosis is a common complication after the arterial switch operation (ASO) for transposition of the great arteries (TGA). Four-dimensional flow (4D flow) CMR provides the ability to quantify flow within an entire volume instead of a single plane. The aim of this study was to compare PA maximum velocities and stroke volumes between 4D flow CMR, two-dimensional phase-contrast (2D PCMR) and echocardiography. A prospective study including TGA patients after ASO was performed between December 2018 and October 2020. All patients underwent echocardiography and CMR, including 2D PCMR and 4D flow CMR. Maximum velocities and stroke volumes were measured in the main, right, and left PA (MPA, LPA, and RPA, respectively). A total of 39 patients aged 20 ± 8 years were included. Maximum velocities in the MPA, LPA, and RPA measured by 4D flow CMR were significantly higher compared to 2D PCMR (p < 0.001 for all). PA assessment by echocardiography was not possible in the majority of patients. 4D flow CMR maximum velocity measurements were consistently higher than those by 2D PCMR with a mean difference of 65 cm/s for the MPA, and 77 cm/s for both the RPA and LPA. Stroke volumes showed good agreement between 4D flow CMR and 2D PCMR. Maximum velocities in the PAs after ASO for TGA are consistently lower by 2D PCMR, while echocardiography only allows for PA assessment in a minority of cases. Stroke volumes showed good agreement between 4D flow CMR and 2D PCMR.

## Introduction

Transposition of the great arteries (TGA) is a common cyanotic congenital heart defect (CHD), accounting for 5–8% of all CHD [1]. In TGA, the aorta arises from the right ventricle (RV) and the pulmonary artery from the left ventricle, for which the arterial switch operation (ASO) combined with the LeCompte manoeuvre is standard of care [2]. Although the ASO results in an excellent survival rate, frequent complications occur such as dilation of the ascending aorta and pulmonary artery stenosis (PS) [2]. Branch PS is the most common cause for reintervention after ASO, with an incidence of up to 20% of ASO patients [2, 3]. Stretching of the pulmonary arteries with the LeCompte manoeuvre, dynamic systolic compression due to the close anatomical relationship with an often dilated ascending aorta, scar formation at the anastomosis site, and atherosclerosis due to altered shear stress distribution are all thought to cause PS after ASO [4]. Frequent and robust non-invasive evaluation of the pulmonary arteries is therefore pivotal for appropriate follow-up of these patients.

Standard non-invasive hemodynamic evaluation of the pulmonary arteries after ASO is currently performed with Doppler echocardiography and two-dimensional phase-contrast cardiovascular magnetic resonance (2D PCMR) [5]. Flow assessment with 2D PCMR relies on measurements from a single fixed imaging plane and thus may not give an accurate representation of the peak velocity or flow volume in the vessel if not appropriately aligned with the blood vessel. Although echocardiography has the benefit of a high temporal resolution for peak velocity measurements, it is greatly dependent on the acoustic window, which can make visualization of the branch pulmonary arteries challenging or even impossible, especially in older children and adults.

Four-dimensional flow (4D flow) CMR provides the opportunity for quantification of flow in an entire volume throughout the complete cardiac cycle. Previous studies have demonstrated 4D flow CMR is a reliable tool for flow and velocity measurements [6, 7]. 4D flow CMR may therefore provide a more comprehensive evaluation of the presence and severity of local or even multilevel PS than can be obtained with Doppler echocardiography or 2D PCMR, as the complete region of the pulmonary arteries can be assessed within a single imaging session. The aim of this study was therefore to compare maximum velocities and flow volumes measured by 4D flow CMR with 2D PCMR and Doppler echocardiography in TGA patients after ASO.

## Methods

### Population

For this study TGA patients after ASO aged 8 to 40 years were prospectively recruited between December 2018 and September 2020. Exclusion criteria included presence of a stent in the pulmonary arteries, presence of a cardiac pacemaker and all contra-indications for CMR including claustrophobia and pregnancy. Patients underwent CMR according to the routine TGA protocol (including cine images) of our centre, with the addition of 4D flow CMR. Routine echocardiography was preferably performed on the same day of CMR. Written informed consent was obtained for all patients and/or their guardians (for patients < 16 years of age). This study was approved by the local Medical Ethical Committee (Study Number 18-200).

### CMR Acquisition

CMR imaging was performed on a 3.0 Tesla scanner (Ingenia R5.6.1, Philips Healthcare, Best, The Netherlands). Velocity encoded 2D PCMR scans with ECG-triggering and a single breath-hold were acquired for the main PA (MPA), left PA (LPA) and right PA (RPA). The plane was positioned at the site where the vessel diameter was considered smallest, which was assessed visually on axial and coronal views. Imaging parameters for the 2D PCMR were as follows: spatial resolution = 1.25 × 1.25 mm^2^, FOV = 320 × 320 mm^2^, slice thickness: 5 mm, number of cardiac phases: 25, echo time = 2.8–3.4 ms, repetition time = 4.9–5.5 ms, flip angle = 10°, bandwith = 479 Hz/pixel, venc = 180–350 cm/s. Scan times were typically around one minute per scan. All scans were checked for velocity-aliasing directly after the end of each scan and repeated with altered venc if necessary.

4D flow CMR acquisition was performed with prospective ECG and respiratory navigator-gating. The acquired volume covered the entire MPA, LPA and RPA. Imaging parameters for the 4D flow CMR were as follows: spatial resolution = 2.5 × 2.5 × 2.5 mm^3^, FOV = 300 × 300–350 × 350 mm^2^, temporal resolution = 32.8–46,1 ms, echo time = 2.1–2.5 ms, repetition time = 3.9–4.5 ms, flip angle = 10°, venc = 200–450 cm/s, TFE factor 3, SENSE: 2.5 (AP) and 1.5 (RL). Concomitant gradient correction and local phase correction was performed from standard available scanner software. Scan times were typically 8–12 min per scan.

### CMR Post Processing

Post processing for 2D PCMR acquisitions was performed with 2D PCMR software (CAAS MR Solutions, version 5.0-5.1, Pie Medical Imaging, Maastricht, the Netherlands). The region of interest was manually segmented by one observer (EW). From these regions of interest, peak velocity, forward flow and regurgitant flow were collected. Stroke volume was defined as forward flow–regurgitant flow and calculated for the MPA, LPA, and RPA.

4D flow CMR data was pre-processed using automatic background and velocity aliasing correction (CAAS MR Solutions, version 5.0-5.1, Pie Medical Imaging, Maastricht, the Netherlands). If aliasing artefacts could not be corrected, patients were excluded from this study. In case of minimal aliasing (defined as one or two voxels) the measurements were performed in the next plane without artefacts. Segmentation of the vessel was performed automatically and subsequent manual correction was done by a single observer with two years of experience in arterial segmentation of 4D flow CMR scans (EW). Regions with the maximum velocity were determined by retrospectively placement of 2D planes at the site where the maximum velocity was suspected, which was determined visually using color-coded streamlines visualization and velocity overlay for the region of interest within the plane. The plane was repositioned until the region with the maximum velocity was identified. From the regions of interest, peak velocity, forward flow and regurgitant flow were collected and stroke volumes were calculated. Aliasing correction was validated using flow mapping in a region proximal and distal to the plane and comparing the flows to the flow in the plane of interest.

### Echocardiography

Echocardiography was performed by an experienced cardiac sonographer using General Electric (GE Healthcare, Wauwatosa, Wisconsin, USA) ultrasound systems, using the optimal transducer for patient size. Parameters collected for this study were maximum instantaneous velocities from Doppler images for the MPA, LPA and RPA.

### Statistical Analyses

Statistical analysis was performed using R version 3.6.3 [8], and figures were produced using the package ggplot2 [9]. All data were assessed for normality using histograms, QQ-plots and the Shapiro–Wilk test. The paired Student’s T-test or Wilcoxon matched-paired signed rank test was used to compare measurements from the different modalities, depending on data distribution (normal or non-normal). Agreement between the different modalities was assessed using Bland–Altman analyses. To assess the proportion of patients with PS in our cohort, we dichotomized patients into two groups based on the peak velocities measured in the RPA and LPA. A peak velocity > 250 cm/s was classified as clinically relevant PS; a lower peak velocity was considered to be normal. The significance level was set at 0.05.

## Results

A total of 45 patients were included between December 2018 and October 2020. Four patients were excluded from analysis due to insufficient quality of the 4D flow CMR acquisition, including severe aliasing which could not be corrected. Two patients were excluded due to severe aliasing in the 2D PCMR scan. Thus, data for 39 patients were analysed with a mean age of 20 ± 8 years. Median age at ASO was 8 (IQR 7–12) days. The most common concomitant cardiac defect was a ventricular septal defect, present in 10 (26%) patients. One patient had undergone aortic valve replacement for severe aortic valve regurgitation and one had surgery for branch PA stenosis. All baseline characteristics are presented in Table 1.

**Table 1:** Baseline characteristics

Characteristic	
Age (years)	20 ± 8
Male	31 (79%)
Height (cm)	171 ± 16
Weight (kg)	64 ± 22
Body mass index (kg/m^2^)	22 ± 5
Age at arterial switch operation (days)	8 (7–12)
Concomitant cardiac defect	
Aberrant coronary artery	2 (5%)
Atrial septal defect	4 (10%)
Bicuspid aortic valve	1 (3%)
Coarctation of the aorta	4 (10%)
Hypoplastic aortic arch	1 (3%)
Ventricular septal defect	10 (26%)
Reintervention	
Aortic valve replacement	1 (3%)
Pulmonary artery plasty	1 (3%)
None	37 (95%)

Data are presented as number (percentage), mean ± standard deviation or median (interquartile range)

Median time between CMR and echocardiography examinations was 20 (IQR 0–69) days. For Doppler echocardiography, velocity measurements of the MPA were not available for 18 patients, in 24 patients for the RPA, and in 23 patients for the LPA. CMR and echocardiography demonstrated preserved biventricular function in all but two patients (LVEF was 49% in these two patients), with a mean left ventricular ejection fraction of 56 ± 5% and a mean RVEF of 56 ± 5% on CMR, and a mean Tricuspid Annular Plane Systolic Excursion (TAPSE) of 19 ± 3 mm on echocardiography. All data on biventricular function is presented in Table 2.

**Table 2:** Cardiac magnetic resonance and echocardiography parameters on biventricular function

Imaging parameter	
LVEDV_i_ (ml/m^2^)	108 ± 25
LVESV_i_ (ml/m^2^)	48 ± 14
LVSV_i_ (ml/m^2^)	60 ± 13
LVEF (%)	56 ± 5
LVCO_i_ (L/m^2^)	4.3 ± 0.8
RVEDV_i_ (ml/m^2^)	102 ± 19
RVESV_i_ (ml/m^2^)	45 ± 11
RVSV_i_ (ml/m^2^)	60 ± 18
RVEF (%)	56 ± 5
RVCO_i_ (ml/m^2^)	4.1 ± 0.7
TAPSE (mm)	19 ± 3

Data are presented as mean ± standard deviation

*I* indexed for body surface area, *LVCO* left ventricular cardiac output, *LVEDV* left ventricular end-diastolic volume, *LVEF* left ventricular ejection fraction, *LVESV* left ventricular end-systolic volume, *LVSV* left ventricular stroke volume, *RVCO* right ventricular cardiac output, *RVEDV* right ventricular end-diastolic volume, *RVEF* right ventricular ejection fraction, *RVESV* right ventricular end-systolic volume, *RVSV* right ventricular stroke volume, *TAPSE* Tricuspid Annular Plane Systolic Excursion

**Fig. 1:**
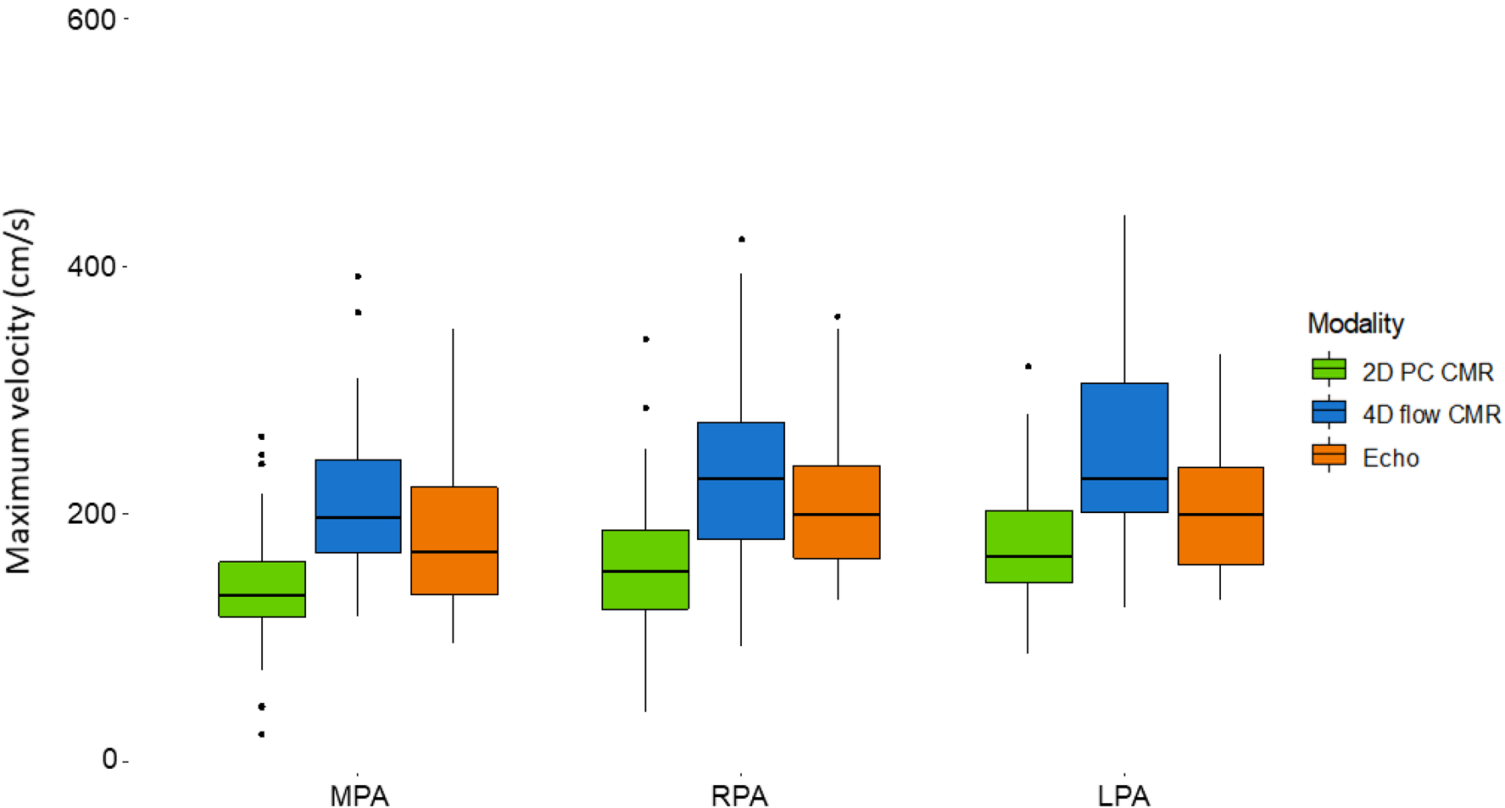
Comparison of maximum velocities in the main, right, and left pulmonary artery as measured by two-dimensional flow phase-contrast cardiac magnetic resonance, four-dimensional flow cardiac magnetic resonance and Doppler echocardiography. *2D PC CMR* two-dimensional phase-contrast cardiac magnetic resonance, *4D flow CMR* four-dimensional flow cardiac magnetic resonance, *MPA* main pulmonary artery, *LPA* left pulmonary artery, *RPA* right pulmonary artery

**Fig. 2:**
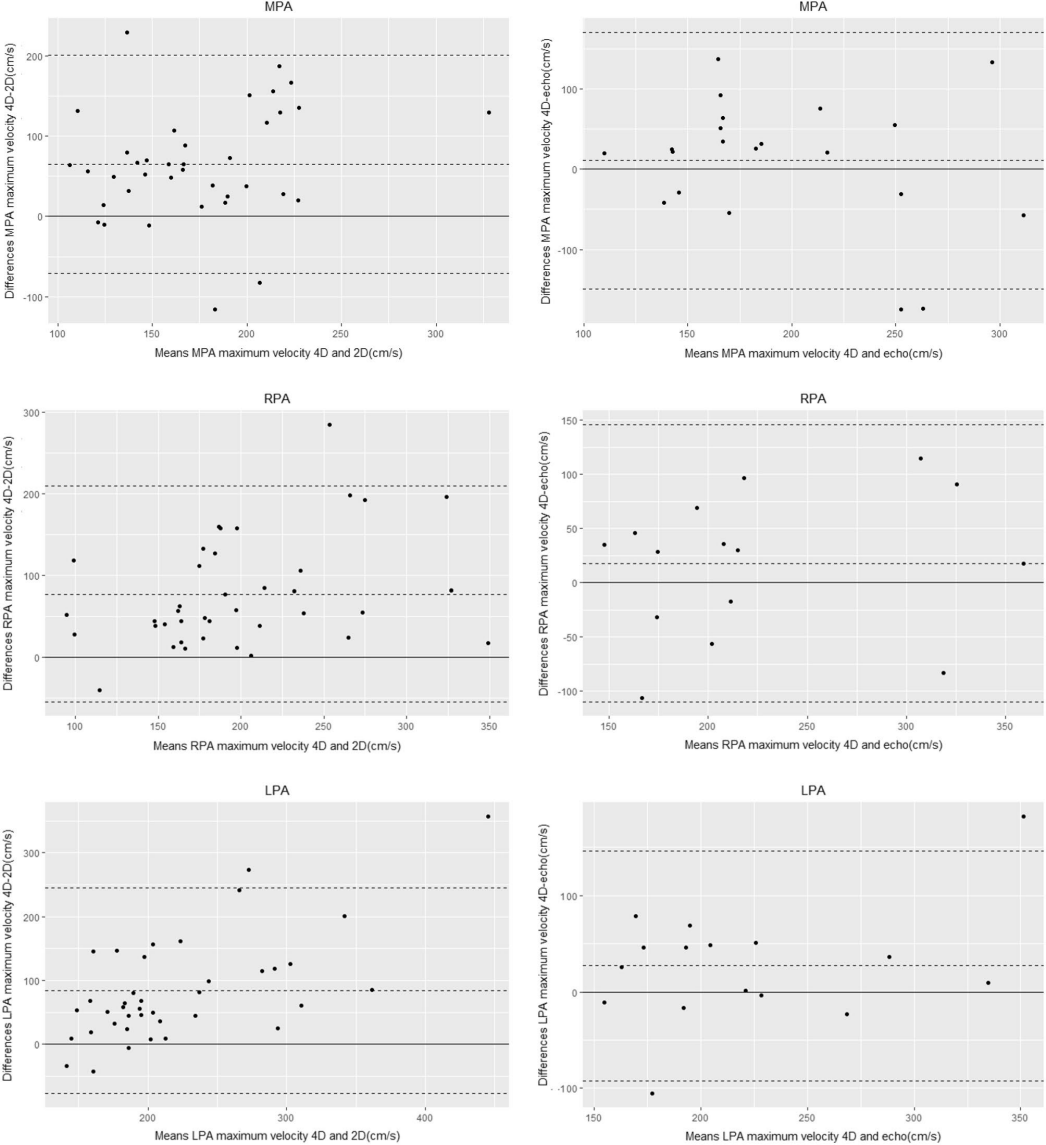
Agreement between four-dimensional flow cardiac magnetic resonance and two-dimensional phase-contrast cardiac magnetic resonance for measurement of maximum velocities in the main, right, and left pulmonary artery (left) and agreement between four-dimensional flow cardiac magnetic resonance and Doppler echocardiography of maximum velocities in the main, right, and left pulmonary artery (right). *2D* two-dimensional phase-contrast cardiac magnetic resonance, *4D* four-dimensional flow cardiac magnetic resonance, *echo* doppler echocardiography, *MPA* main pulmonary artery, *LPA* left pulmonary artery, *RPA* right pulmonary artery

Maximum velocities as measured by 4D flow CMR were significantly higher when compared to 2D PCMR measurements in the MPA, RPA and LPA (p < 0.001 for all, respectively) (Table3, Fig. 1). There was no significant difference between maximum velocities as measured by 4D flow CMR and by Doppler echocardiography (Table 4, Fig. 1). Bland–Altman plots (Fig. 2) showed 4D flow CMR peak velocity measurements were consistently higher than those by 2D PCMR with a mean difference of 65 cm/s for the MPA and a mean difference of 77 cm/s for both the RPA and LPA. Bland–Altman plots comparing peak velocity measurements by 4D flow CMR and echocardiography (Fig. 2) showed good agreement with a mean difference of 11 cm/s for the MPA, 18 cm/s for the RPA, and 27 cm/s for the LPA.

**Table 3:** Comparison of maximum velocities between four-dimensional flow cardiac magnetic resonance and two-dimensional phase contrast cardiac magnetic resonance

Location	Peak velocity 4D (cm/s)	Peak velocity 2D (cm/s)	p-value
MPA	197 (169–244)	135 (118–162)	< 0.001
RPA	229 (180–274)	154 (124–188)	< 0.001
LPA	230 (201–305)	166 (145–203)	< 0.001

Data are presented as median (interquartile range)

*2D* two-dimensional, *4D* four-dimensional, *MPA* main pulmonary artery, *LPA* left pulmonary artery, *RPA* right pulmonary artery

**Table 4:** Comparison of maximum velocities between four-dimensional flow cardiac magnetic resonance and Doppler echocardiography

Location	Peak velocity 4D (cm/s)	Peak velocity echo (cm/s)	p value
MPA (n = 21)	195 (154–235)	170 (135–222)	0.394
RPA (n = 15)	224 (187–255)	200 (165–240)	0.277
LPA (n = 16)	226 (173–277)	200 (160–238)	0.065

Data are presented as median (interquartile range)

*2D* two-dimensional, *4D* four-dimensional, *MPA* main pulmonary artery, *LPA* left pulmonary artery, *RPA* right pulmonary artery

We dichotomized patients into two groups based on the maximum velocities measured in the RPA and LPA; a peak velocity > 250 cm/s was classified as clinically relevant PS; a lower peak velocity was considered to be normal. 4D flow CMR measurements identified a substantially higher number of patients with PS than with 2D PCMR measurements. For the LPA, 14 patients (36%) were identified to have PS based on 4D flow CMR measurements, versus only 2 patients (5%) based on 2D PCMR measurements. Similarly, 14 patients (36%) had PS of the RPA based on 4D flow CMR measurements, compared to only 3 patients (8%) based on 2D PCMR measurements.

**Fig. 3:**
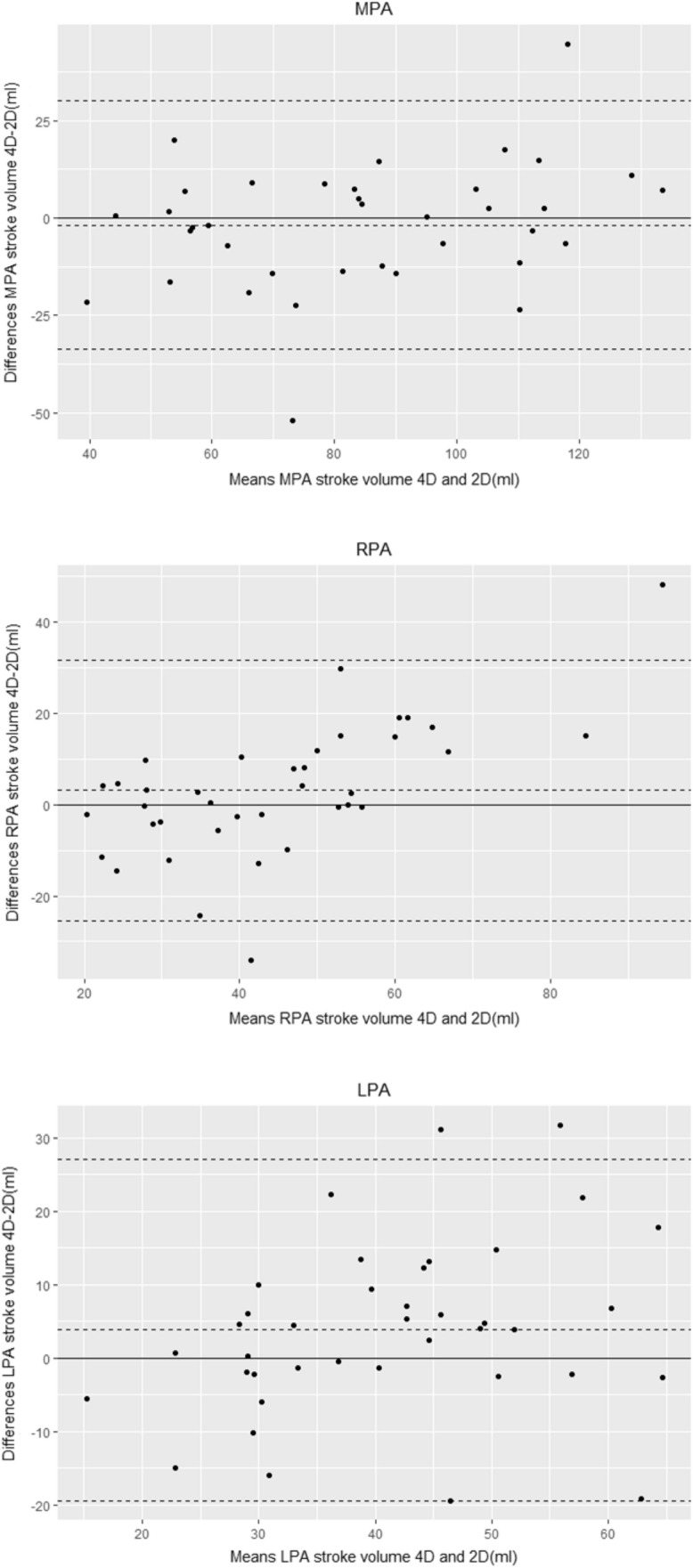
Agreement between four-dimensional flow cardiac magnetic resonance and two-dimensional phase-contrast cardiac magnetic resonance for measurement of stroke volumes (forward flow–regurgitant flow) in the main, right, and left pulmonary artery. *2D* two-dimensional phase-contrast cardiac magnetic resonance, *4D* four-dimensional flow cardiac magnetic resonance, *MPA* main pulmonary artery, *LPA* left pulmonary artery, *RPA* right pulmonary artery

Stroke volumes measured by 4D flow CMR were not significantly different when compared to 2D PCMR in the MPA, LPA and RPA. (Table 5). Bland–Altman plots (Fig. 3) show good agreement between 4D flow CMR and 2D PCMR measurements of flow volumes, with a mean difference of 1.8 ml for the MPA, 3.1 ml for the RPA, and 3.8 ml for the LPA.

**Table 5:** Comparison of stroke volumes (forward–regurgitant flow) between four-dimensional flow cardiac magnetic resonance and two-dimensional phase-contrast cardiac magnetic resonance

Location	Stroke volume 4D (ml)	Stroke volume 2D (ml)	p-value
MPA	85 ± 29	85 ± 24	0.503
RPA	46 ± 22	43 ± 13	0.196
LPA	48 ± 24	43 ± 13	0.102

Data are presented as mean ± standard deviation

*2D* two-dimensional phase-contrast CMR, *4D* four-dimensional flow CMR, *MPA* main pulmonary artery, *LPA* left pulmonary artery, *RPA* right pulmonary artery

## Discussion

The goal of the present study was to compare maximum velocities and stroke volumes in the PAs between 4D flow CMR, 2D PCMR and Doppler echocardiography and conveys the following findings:

1. Maximum velocities measured by 4D flow CMR are significantly higher compared to maximum velocities measured by 2D PCMR, but similar to maximum velocities by Doppler echocardiography.
2. Maximum velocities in the MPA and branch PAs could be evaluated in almost all TGA patients. In contrast, the branch PAs for the majority of TGA patients could not be visualized using echocardiography.
3. Stroke volumes measured by 2D PCMR were not significantly different compared to stroke volumes measured by 4D flow CMR for the MPA and branch PAs.

We found maximum velocities measured by 4D flow CMR to be significantly higher than measured by 2D PCMR. There are several reasons for the underestimation of the maximum velocities by conventional 2D PCMR. First, the positioning of the 2D imaging planes was done based on visual assessment of the PAs and placed where the diameter was considered to be the narrowest. The peak velocity can only be measured in that specific plane, whereas 4D flow CMR provides the opportunity to measure maximum velocities along the entire length of the pulmonary vessel. Second, the 2D PCMR plane is positioned by the operator based on 2D anatomical images and flow is measured in one direction: orthogonal to this plane. When the plane has not been positioned exactly perpendicular to the vessel, this can give an underestimation of the velocity magnitude [10, 11]. With 4D flow CMR, the plane for analysis can be positioned retrospectively and with use of three-dimensional anatomical data and visualization of the flow to ensure the plane is positioned exactly perpendicular to the vessel and the blood flow. Last, since 2D PCMR only measures flow in one direction, it does not take into account turbulent flow, which is often present in patients with CHD. With the three-dimensional velocity-encoding of 4D flow CMR, eccentric flow can be taken into account, resulting in higher maximum velocities [12, 13].

To our knowledge, only one prior study compared maximum velocities as measured by 4D flow CMR, 2D PCMR and Doppler echocardiography in patients after the arterial switch operation [12]. Jarvis et al. found significantly higher velocities using 4D flow CMR in the MPA and RPA, but not in the LPA, and no difference in maximum velocities between Doppler echocardiography and 4D flow CMR. Their results are thus partially in line with our results. However, there are important differences between our study population and theirs. Our population was considerably older: 20 ± 8 years (range 8–37 years) versus 13 ± 9 years (range 1–25 years) and we most likely included more patients with branch PS, as comparison of maximum velocities in our study versus Jarvis et al. revealed 2.1 ± 0.8 m/s versus 1.8 ± 0.6 m/s for the RPA and 2.4 ± 1.0 m/s versus 1.7 ± 0.5 m/s for the LPA. Since visualization of PAs on echocardiography becomes more difficult with increasing age, 4D flow CMR is especially suitable for older children and adults.

To assess the clinical impact of the hemodynamic evaluation of 4D flow CMR versus 2D PCMR, we analysed the proportion of patients that would be classified as having PS in our centre based on peak velocity as measured by both modalities. When using 250 cm/s as the cut-off value for the diagnosis of substantial PS, we found an increase in the proportion of patients with PS when comparing 2D PCMR with 4D flow CMR. Since there is no literature available on the cut-off value of significant stenosis in these patients, we chose the cut-off value generally used in our centre. Due to the lack of evidence on cut-off values Jarvis et al. decided not to perform such an analysis [12]. Therefore, these results need to be interpreted with caution. Furthermore, since evidence for intervention for PS in older children and adults is lacking, the impact of these findings on (re)intervention in this patient group warrants further investigation.

We found no significant differences between maximum velocities measured by 4D flow CMR and Doppler echocardiography. However, in the majority of patients the PAs could not be visualized using echocardiography. It is well known the acoustic window severely limits the ability to visualize PAs in older children and adults, especially in patients after ASO, with a retrosternal position of the PAs [14]. Echocardiography is often the imaging modality of choice for follow-up of these patients due to it being widely available, cost-effective and non-invasive. Based on the results of this study, 4D flow CMR should be considered when imaging quality of echocardiography is insufficient.

We found no significant difference in stroke volumes in the main and branch PAs when comparing 4D flow CMR and 2D PCMR. Our results are in line with a previous study by Nordmeyer et al. in healthy volunteers and CHD patients, in which no differences were found when comparing flow volumes measured by 4D flow CMR and 2D PCMR [15].

In general, 4D flow CMR has important advantages over 2D PCMR for the evaluation of PS in patients after ASO: the ability to position planes of interest exactly perpendicular to the vessels at any point within the scanned volume, the fact that it has velocity encoding in all three spatial directions and the ability to visualize the blood flow. We believe that 4D flow CMR should be considered as an important tool in the hemodynamic assessment of TGA patients after ASO, given the clear need for comprehensive serial evaluation of the cardiovascular system.

## Limitations

There are several limitations that need to be taken into account for this study. First, the CMR and echocardiography were not always performed on the same day. We included echocardiography up to one year prior to CMR to limit the effect of development of PS over time. Second, 4D flow CMR has a limited spatial and temporal resolution, a relatively long acquisition time and time and skill required for post-processing [16]. These limitations currently hamper widespread clinical implementation for the follow-up of patients after ASO, although recent improvements in CMR techniques have provided shorter acquisition times and more user-friendly postprocessing software.

## Conclusion

This study shows that 4D flow CMR detects higher maximum velocities in the PAs of TGA patients when compared to 2D PCMR, while echocardiography only allows for PA assessment in a minority of cases. In our cohort, a substantial number of patients would be classified as having PS based on 4D flow CMR measurements, in contrast to 2D PCMR measurements. No differences were found between stroke volumes in the PAs measured by 4D flow CMR compared to 2D PCMR.

## Data Availability

The data that support the findings of this study are available on request from the corresponding author.

## Abbreviations

2D: Two-dimensional
4D flow: Four-dimensional flow
ASO: Arterial switch operation
CHD: Congenital heart disease
CMR: Cardiac magnetic resonance
LPA: Left pulmonary artery
MPA: Main pulmonary artery
PCMR: Phase contrast cardiac magnetic resonance
PA: Pulmonary artery
PS: Pulmonary artery stenosis
RPA: Right pulmonary artery
TAPSE: Tricuspid annular plane systolic excursion
TGA: Transposition of the great arteries
